# Public satisfaction with health system after healthcare reform in China

**DOI:** 10.1186/s12961-023-01067-6

**Published:** 2023-12-04

**Authors:** Lili Kang, Tianyi Zhang, Bensong Xian, Changle Li, M. Mahmud Khan

**Affiliations:** 1 School of Health Management, Fujian Medical University, Fuzhou, 350122 Fujian China; 2 School of Basic Medical Sciences, Shandong University, Jinan, China; 3 School of Health Management, Inner Mongolia Medical University, Hohhot, China; 4 Department of Health Policy and Management, College of Public Health, University of Georgia, Athens, GA United States of America

**Keywords:** Public satisfaction, Health system, Perceived quality of care, China

## Abstract

**Background:**

The Chinese central government launched the third phase of health system reforms in 2009. After a decade since the initiation of the reform, the health system has witnessed noteworthy gains. However, there is no concurrent improvement in public satisfaction with the health system. This study analysed various factors that influence public satisfaction with the system and examined whether perceived quality of care affects public satisfaction.

**Methods:**

A longitudinal nationally representative survey was used for this study. We used five waves of China Family Panel Studies (CFPS) survey data. The final sample consisted of 145 843 observations. A two-way fixed-effects ordered logistic model was used for the analysis.

**Results:**

The results indicate that perceived good quality of care was positively associated with public satisfaction in health system regardless of rural–urban residence. Older adults and individuals with more than 3 years of college education were less likely to be satisfied with the system in rural areas. Personal income and the density of medical professionals in the geographic area tend to improve public satisfaction in rural areas. Having medical insurance coverage and fair or good self-rated health improved the probability of reporting public satisfaction in urban areas. Married people and individuals who lived in the West region were less likely to be satisfied with the health system in urban areas.

**Conclusions:**

Knowledge and skills of healthcare providers or physical quality of facilities are not sufficient in improving public satisfaction in the health system. Policymakers need to identify options to influence the important factors that affect public perception of the system. This analysis identified several policy-amenable factors to improve public perception of the health system in rural and urban China.

## Background

China’s rapid economic growth following its shift from a central planning to a socialistic market economy in 1978 was also accompanied by three major health system reforms. The first round of reforms (1978–2022) marked the beginning of adopting a healthcare funding strategy that is based on laissez faire market forces. Public medical institutions, for financial sustainability, had to charge patients [1]. The changes in economic structure triggered by market economy policies had adverse effects on the commune-based healthcare safety net (Rural Cooperative Medical Scheme) in the 1980s, and the number of uninsured rural residents saw a dramatic increase [2]. In urban areas, state-owned enterprises faced market competition without government subsidies. Labor Medical Service Scheme (LMSS) encountered difficulties such as poor capacity of risk pooling and rising healthcare costs. LMSS was replaced with a new healthcare insurance called the Basic Medical Insurance for Urban Employees (BMIUE) in 1998 [3].

Lack of insurance coverage drove the second round of reforms in China (2002–2008) [1]. In 2003, the Chinese government launched a medical mutual assistance scheme called the New Rural Cooperative Medical Scheme (NRCMS) for rural residents, receiving funding from the central government, local government and individuals [4], and 4 years later, the government established a similar medical insurance scheme called the Basic Medical Insurance for Urban Residents (BMIUR), which covered urban residents not covered by the BMIUE [1]. Although insurance coverage has increased rapidly, access to and quality of healthcare, disparity in availability of healthcare facilities between urban and rural areas and inefficiencies in health system became significant concerns in China [1].

Since the launch of the third round of health system reforms in 2009, the Chinese government has made considerable investments in primary care facilities and issued a series of new policies to expand insurance coverage, reforming the pharmaceutical market and pilot-testing public hospital reforms [5]. The objective of the new health reform was to establish an equitable and effective health system for all people by 2020 [6]. After nearly a decade since the reforms, the reforms have made many noteworthy gains: they increased public funding for health, achieved near-universal health insurance coverage, improved access to healthcare, and decreased health inequality [6, 7]. These policies should have improved public satisfaction with the health system as well, but no significant increase in satisfaction has been observed [8].

Public satisfaction with the health system is the degree to which the population, in general, report satisfaction with the health system [9]. Public satisfaction is a broader concept than patient satisfaction, reflecting the general population’s interaction and experience with the health system and the extent to which the system meets their expectations [10, 11]. Lower expectations lead to higher public satisfaction and vice versa [12]. Unlike patient satisfaction, which focusses on users of health services, the subjective measure of public satisfaction includes opinions of all, that is, both non-users and users of health services [13], which is a critical indicator of health system performance [11]. The degree of public satisfaction is important for policy and decision-makers and is a significant aspect to consider when reforming the health system.

Public satisfaction is assessed through survey questions using a self-reported Likert scale rating [14]. In 1988, Louis Harris and Associates assessed public satisfaction using a single question about their overall view of the health system (minor changes needed, fundamental changes needed and completely rebuild the system) [15, 16]. The International Health Policy Survey also asked respondents about their view of the health system [17]. In 1996, the Eurobarometer survey used the following question: ‘In general, would you say you are very satisfied, fairly satisfied, neither satisfied nor dissatisfied, fairly dissatisfied or very dissatisfied with the way health care runs in your country?’ to collect information on public satisfaction [18]. Since then, many surveys have employed a similar question to assess public satisfaction of health systems, such as the World Health Survey, the Chinese General Social Survey and the Living Conditions, Lifestyles, and Health Study [11, 19, 20]. In addition, the level of confidence in receiving medical care from the health system and the severity of the nation’s healthcare problem are also used to evaluate public satisfaction [8, 21].

Most of the studies on public satisfaction with the health system were carried out in high-income countries, especially in the United States and European countries [10, 15–20], and a few studies were conducted in low-income and middle-income countries, such as China, Ghana and Brazil [11, 22, 23]. Review of the literature implies that a host of factors affect public satisfaction with the health system. Socio-demographic characteristics affecting public satisfaction include age, gender, place of residence, educational attainment, household economic status and employment status [9–11, 13, 19, 20, 22]. The findings are mixed on how age, educational attainment and employment status affect public satisfaction. For example, Bleich et al. [19] reported that older persons are more likely to be satisfied with the health system. In contrast, Footman et al. [20] found that individuals are more likely to be satisfied with the health system with decreasing age. Zhang et al. [11] showed that higher educational attainment is more likely to be satisfied with the health system. However, Yip et al. [9] found that there is no significant association between educational attainment and public satisfaction. Amoah et al. [22] found that employed persons show a higher likelihood of being satisfied with the health system, while Peng and Zhang [24] reported the opposite result.

Effects of insurance coverage and health status of population also affect public satisfaction. For example, Yip et al. [9], Munro et al. [13], and Hero et al. [25] found that insured persons report higher satisfaction with the health system compared with those who were not insured. Bleich et al. [19], Footman et al. [20] and Zhang et al. [11] found that people with poor health are less likely to be satisfied with the health system. These three population-based cross-sectional studies were conducted in 21 European Union countries, 9 former Soviet Union countries and China, and logistic regression and ordinary least squares regression models were used to assess the association between health status and public satisfaction. In addition, previous studies found positive associations between public satisfaction and trust in the health system or political institutions, attitudes towards welfare policies and ideological beliefs (egalitarian) [13, 20, 22, 26].

Association between public satisfaction and government spending on healthcare is mixed. For example, Zhang et al. [11] found that there is a negative association between public satisfaction and government expenditures as percent of total healthcare expenditure. Yuan [27] showed that higher public spending on healthcare is associated with higher satisfaction with the health system. Furthermore, previous studies have shown that media use, expectations from medical care, and availability of healthcare resources also affect the degree of public satisfaction [11, 13, 17, 27–29]. Patient’s experiences with the health system are a significant factor in public satisfaction as well, which explains, according to some studies, approximately 10% of the variation of public satisfaction measures [19, 27].

Patient satisfaction attempts to capture patient perceptions of the quality of health services delivered by a health provider [30]. Previous research studies have shown positive associations between patient satisfaction and perceived quality of care [31–33]. Given the importance of quality of care in improving health outcomes and wellbeing, it is assumed that improving the quality of health services ought to be a priority for any health system [34]. However, evidence on associations between public satisfaction and perceived quality of care is limited. To fill these research gaps, the objectives of this study are (1) to identify the factors associated with public satisfaction with the health system using a nationally representative, longitudinal survey; and (2) to examine the association between perceived quality of care and public satisfaction with the health system.

## Methods

### Study design

The study design used in the study is a longitudinal study.

### Study setting

**Fig. 1:**
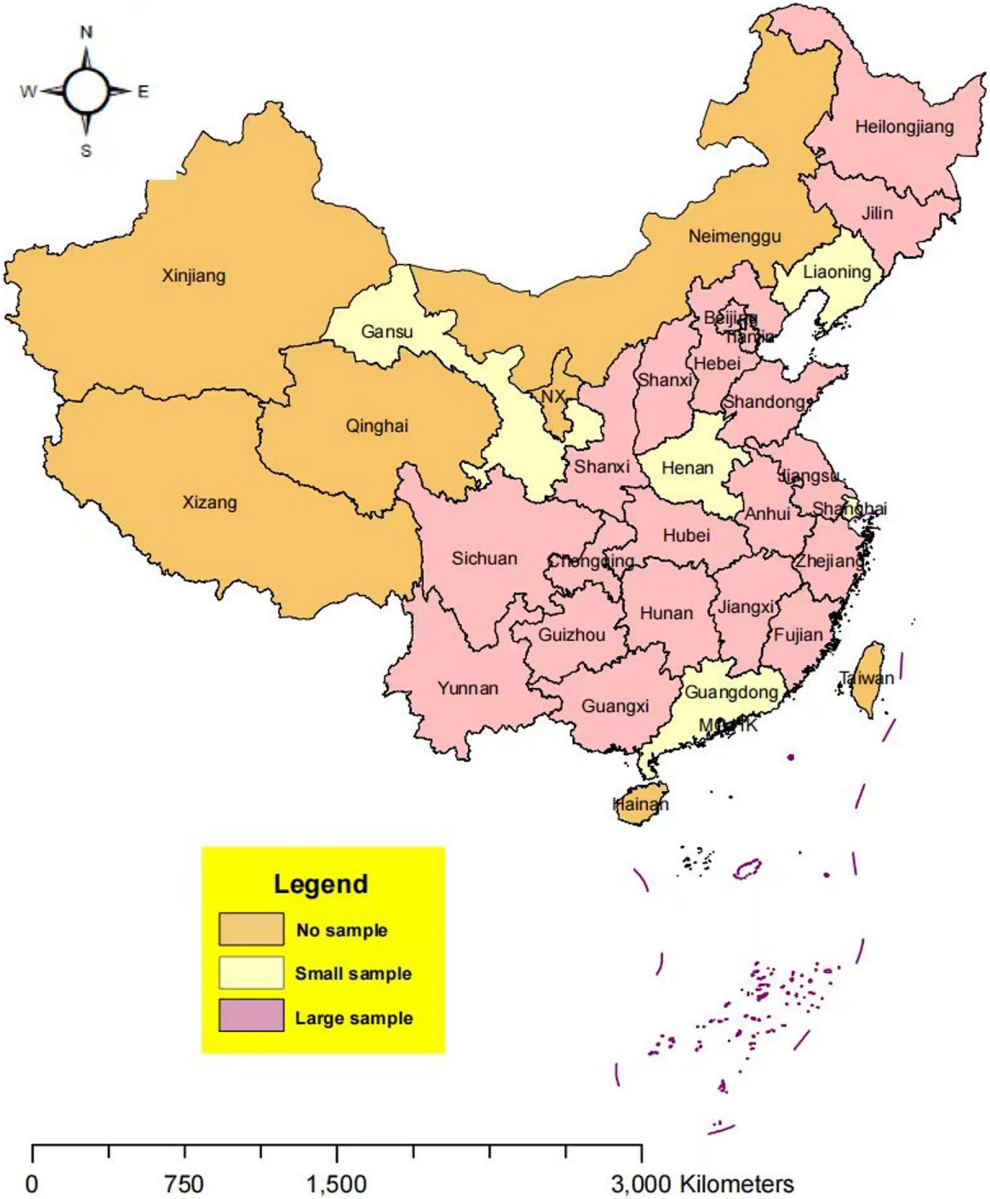
Representativeness of the samples

The data used in this study were obtained from five waves of panel data from the China Family Panel Studies (CFPS 2012, 2014, 2016, 2018 and 2020). The CFPS is a nationally representative, biennial survey initiated by the Institute of Social Science Survey of Peking University in 2010. A multistage probability sample proportional to size was used to select and interview households covering 25 provinces and their administrative equivalents. Five large provinces or municipalities (Gansu, Guangdong, Henan, Liaoning and Shanghai) were selected to oversample populations, and the remaining 20 provinces (or administrative equivalents) were grouped as a large province (see Fig. 1). Each of the six CFPS subsamples was selected through three stages: (1) county or their administrative equivalent, (2) village (rural areas) or resident committee (urban areas) and (3) household. The CFPS interviewed almost 15 000 families and more than 42 000 individuals within these families, including children and adults. The CFPS sample is representative of the 25 provinces in China once the proper sample weights are used, thereby representing the entire Chinese population. The CFPS represents 94.5% of the total population in the Chinese mainland [35].

The CFPS primarily conducts face-to-face interviews aided by computer-assisted personal interviewing (the interviewer uses a tablet to record answers given during the interview), starting in July and ending in June of the following year. When the CFPS fails to complete face-to-face interviews, telephone or web-based interviews are used as a substitute. The 2020 CFPS was mainly conducted through telephone interviews due to the coronavirus disease 2019 (COVID-19) pandemic and about 89% of respondents were interviewed by telephone. More details on the sampling procedure and data collection process are available in Xie and Hu [36]. The CFPS consists of the following modules: demographics, family structure/transfer, health status and functioning, biomarkers, health care and insurance, work, income and consumption, assets (individual and household) and community-level information. The instruments have high reliability in the CFPS of adults, which encompass areas such as mental health, social capital and parental engagement. The Cronbach’s alpha ranges from 0.61 to 0.85 [37, 38].

### Study population

The CFPS respondents are interviewed every 2 years. In the first wave in 2010, 33 600 adults (older than 16 years) successfully completed full-length questionnaires. The adult sample sizes using full-length questionnaires in subsequent waves of surveys in 2012, 2014, 2016, 2018 and 2020 were 32 159, 31 597, 33 244, 30 593 and 23 048, respectively. The 2010 CFPS survey did not collect information on public satisfaction in healthcare. Therefore, this study created a five-period unbalanced panel dataset from 2012 to 2020. After eliminating all cases with missing relevant data, the final sample consisted of 145 843 observations from 47 397 adults across all five waves of the surveys.

### Theoretical model

**Fig. 2:**
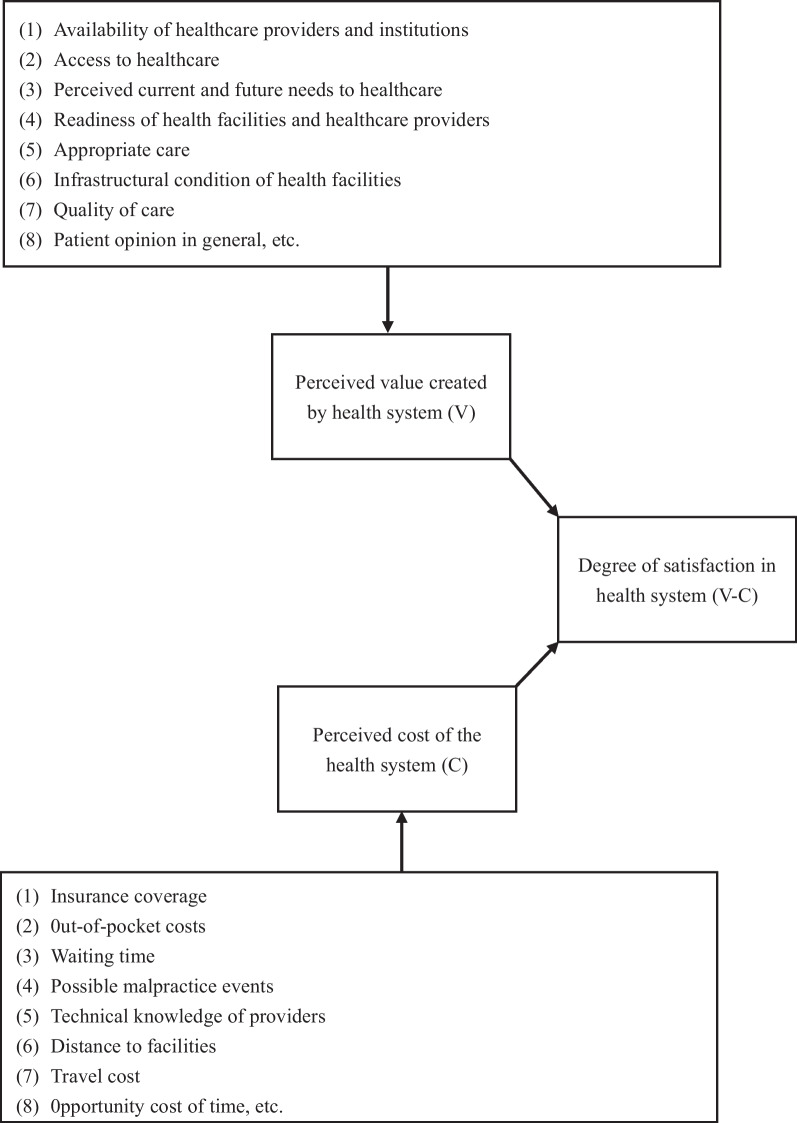
Theoretical model of this study

The current study employed a consumer-centric perspective of value creation. From a consumer-centric perspective, customer value is defined as a process of weighing benefits and costs [39, 40]. In theory, the degree of satisfaction with the health system should be determined by the weighing of the perceived value created by the health system against the perceived cost or concerns associated with the health system. Figure 2 presents the theoretical model of this study.

### Measures

Public satisfaction with the health system was defined as an ordinal dependent variable with values ranging from 0 to 10, with 0 indicating completely dissatisfied and 10 indicating completely satisfied. The CFPS used the following question to obtain the information: ‘How would you rate the degree of severity of China’s healthcare problem?’; with 0 ‘very low severity’ and 10 ‘very high severity’. The severity score for the healthcare problem is a reflection of public satisfaction with the health system because it indicates the respondent’s perception of functioning of the system. The degree of severity of problems as a measure of public satisfaction in the health system has been suggested by several previous studies [8, 41, 42]. The inverse relationship between severity of healthcare complaints and patients’ satisfaction with the system has been documented [43]. In our analysis, we have defined the reporting of ‘high severity of problems’ as being ‘completely dissatisfied’ with the system and assigned a value of 0 for this response. Similarly, if the respondent rated the health system problems as ‘very low severity’, it is considered an indication of high level of public satisfaction (with value assigned as 10).

The present study measured perceived quality of care from two perspectives: provider competence and provider structural quality. Provider competence was grouped into five levels: very bad, bad, fair, good and very good. The question in the CFPS that collected information on provider competence is: ‘How would you evaluate the knowledge, expertise, skills and abilities of the health care provider that you visit most often?’. Provider structural quality was also divided into five categories: very dissatisfied, dissatisfied, fair, satisfied and very satisfied, on the basis of a question that asks: ‘Are you satisfied with the condition of the healthcare facility that you visit most often (such as the adequacy of facilities, equipment, staff and drugs, qualifications of physicians and nurses, administrative structures and convenient transportation)?’.

In the CFPS, respondents were also asked to report on their perceived health status by posing the question, ‘Rate your health status today. Choose your health status from the following health status categories: excellent, very good, good, fair or poor.’ Controlling for response bias in self-rated health status, a balanced set of one positive category (good), one neutral category (fair) and one negative category (poor) were defined [44]. We have defined three health status categories for our empirical analysis: poor, fair and good (excellent, very good and good).

The following four categories of variables were used to explain the variability of public satisfaction with the health system: (1) socio-demographic characteristics: age groups (16–29, 30–39, 40–49, 50–59 and ≥ 60 years old), sex (male), educational attainment levels (illiterate/semi-literate, elementary school, middle school, high school and > 3 years of college), marital status (married), place of residence (urban), personal income, employment status (employed), having medical insurance and geographic area (northeast, east, central and west regions); (2) health status: self-rated health status and having chronic conditions (had doctor-diagnosed chronic diseases in the past 6 months); (3) patient experience: hospitalization history (reported hospitalization); and (4) health system performance: density of hospital beds and medical professionals (per 1000 population) in urban and rural areas of each province and municipality. The data were obtained from the China Health Statistical Yearbooks.

### Statistical analyses

A descriptive analysis of public satisfaction was performed by considering socio-demographic characteristics and perceived quality of care. Statistical significance between groups was assessed through one-way analysis of variance (ANOVA).

A two-way fixed-effects ordered logistic model was used in this study. The empirical model is based on a latent regression and is defined as follows:

$${y}_{it}^{*}={x}_{it}^{\prime}\gamma +{P}_{it}^{\prime}\beta +{\alpha }_{i}+{\delta }_{t}+{\varepsilon }_{it}$$

The vector of covariates *x*_it_ includes socio-demographic characteristics, health status, past clinical experiences, and health system performance as described above. The vector ${P}_{it}^{\prime}$ represents perceived quality of care (provider competence and provider structural quality). $\gamma$ and $\beta$ are the estimated coefficients. The unobserved time-constant, unit-specific confounders (${\alpha }_{i}$) are called the unit fixed-effects and can be correlated with the variables in the model. Furthermore, the unobserved unit-constant, time-specific confounders (${\delta }_{t}$) represent the time fixed-effects. The errors $\varepsilon$ are logistically distributed across observations and standardized at mean of zero and variance of π^2^/3. ${y}_{it}^{*}$ is an unobserved latent variable linked to the observed ordinal response categories public satisfaction with health system (${\mathrm{PSH}}_{it}$).

$${\text{PSH}}_{it}=\left[\begin{array}{ll} 0, & \text{if} \; {y}_{it}^{*}\le {\mu }_{0} \\ 1, & \text{if} \; {\mu }_{0}<{y}_{it}^{*}\le {\mu }_{1}\\ & .\\ & .\\ & .\\ 9, & \text{if} \; {\mu }_{8}<{y}_{it}^{*}\le {\mu }_{9}\\ 10, & \text{if} \; {\mu }_{9}<{y}_{it}^{*}\\ \end{array}\right]$$

where $\mu$ are the underlying thresholds that define the theoretical distribution of the level of PSH, subject to the constraint that $0<{\mu }_{1}<{\mu }_{2}<\cdots {<\mu }_{9}$. The fixed-effects ordered logistic model relies on the parallel-lines assumption, which means the coefficient vectors $\gamma$ and $\beta$ are identical for 11 categories of PSH. The two-way fixed-effects ordered logistic model employed the “blow-up and cluster” estimator from Baetschmann et al. [45, 46]. Since the healthcare delivery system differs between urban and rural areas, the two-way fixed-effects ordered logistic model was used to analyse factors affecting public satisfaction stratified by urban–rural residence, which should help avoid potential bias created by differences between urban and rural health systems. The results are presented as odds ratios (ORs) along with 95% confidence intervals (CIs). All statistical analyses were conducted using Stata Version 17 (StataCorp, College Station, TX).

## Results

**Fig. 3:**
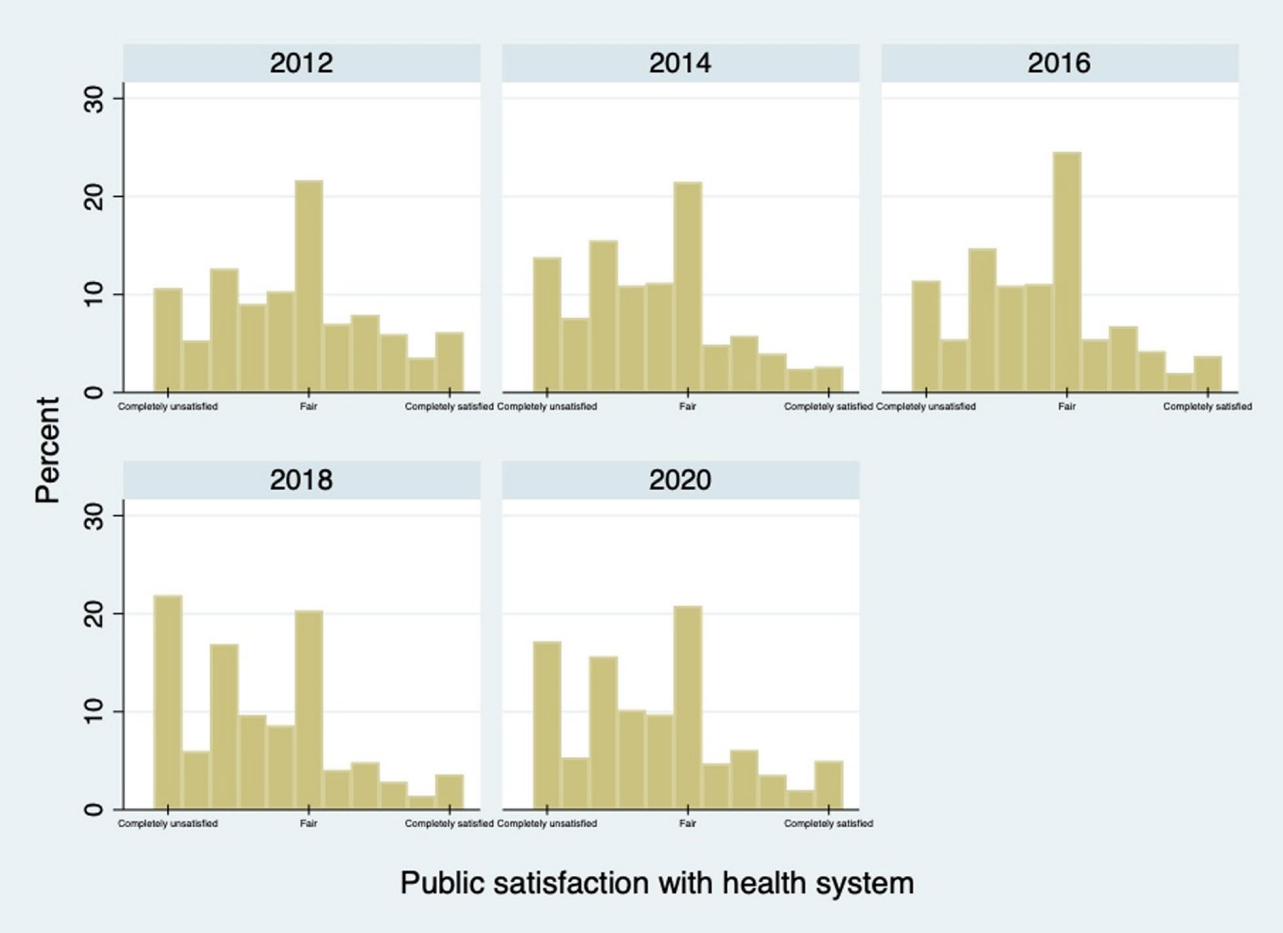
Percentage of persons reporting public satisfaction by 0–10 scale of public satisfaction level in the five waves

A descriptive summary of all variables over time is presented in Table 1. The mean public satisfaction score decreased from 4.45 in 2012 to 3.34 in 2018, and the mean public satisfaction score during the COVID-19 pandemic increased slightly to 3.76. The proportion of persons reporting provider competence being good or very good increased from 37.29% in 2012 to 63.23% in 2020. Slightly more than half of the surveyed individuals reported satisfaction (satisfied or very satisfied) with provider structural quality in 2012. This proportion increased to 77.54% in 2020. Figure 3 shows the distribution of public satisfaction scores with the health system on the 0–10 scale in the five waves of the survey with 95% confidence intervals. The plot of reported values for the 0–10 public satisfaction scale displays a positively skewed distribution, with most respondents reporting public satisfaction in the range 0 to 5 in all five waves. It also shows some lumping of values at 0, 2 and 5 in all five waves.

**Table 1:** Description of the variables over five waves (mean or %)

	2012	2014	2016	2018	2020
	*N* = 30 968	*N* = 30 994	*N* = 31 866	*N* = 29 851	*N* = 22 164
Public satisfaction with health system (mean)	4.45	3.73	4.03	3.34	3.76
Provider competence (%)					
Very bad	0.83	0.87	1.07	1.83	1.74
Bad	5.49	4.79	4.71	10.29	8.62
Fair	56.39	57.45	56.94	33.59	26.40
Good	30.83	28.55	28.16	44.23	48.90
Very good	6.46	8.33	9.13	10.07	14.33
Provider structural quality (%)					
Very unsatisfied	0.55	0.62	0.69	1.53	1.31
Unsatisfied	5.90	5.10	5.66	8.46	6.48
Fair	40.42	43.71	40.91	22.94	14.67
Satisfied	49.43	45.47	47.65	59.30	67.76
Very satisfied	3.70	5.10	5.09	7.77	9.78
Age group (%)					
16–29	21.86	21.42	22.29	19.83	20.42
30–39	15.93	14.82	15.76	16.64	20.42
40–49	22.91	21.59	19.45	17.91	16.90
50–59	18.01	18.49	18.25	19.68	20.46
≥ 60	21.29	23.69	24.24	25.95	21.81
Sex					
Male	49.20	49.10	49.92	49.60	50.21
Female	50.80	50.90	50.08	50.40	49.79
Educational attainment (%)					
Illiterate/semi-literate	28.66	27.08	25.04	22.65	17.52
Elementary school	21.37	21.63	20.69	19.23	18.60
Middle school	28.49	28.89	28.97	29.74	31.34
High school	13.89	14.26	14.69	16.41	17.38
More than 3 years of college	7.59	8.13	10.62	11.98	15.16
Marital status (%)					
Married	79.68	79.45	78.37	78.46	76.85
Other	20.32	20.55	21.63	21.54	23.15
Place of residence (%)					
Urban areas	45.22	47.81	49.17	50.55	51.52
Rural areas	54.78	52.19	50.83	49.45	48.48
Personal income (10 000 RMB) (mean)*	2.37	2.88	3.51	4.00	4.70
Employment status (%)					
Employed	62.06	65.96	65.38	66.29	66.65
Other	37.94	34.04	34.62	33.71	33.35
Medical insurance coverage (%)					
Yes	87.12	91.05	91.07	91.75	90.28
No	12.88	8.95	8.93	8.25	9.72
Locations of respondents (%)					
Northeast region	14.22	14.33	13.53	13.10	12.89
East region	32.62	33.06	32.48	32.77	33.04
Central region	24.99	25.41	24.57	23.69	24.15
West region	28.16	27.20	29.43	30.44	29.92
Self-rated health status (%)					
Poor	18.00	15.39	14.98	16.29	13.73
Fair	18.69	14.24	17.55	13.06	10.01
Good	63.31	70.37	67.47	70.65	76.26
Chronic conditions (%)					
Yes	12.40	16.64	16.31	16.70	14.70
No	87.60	83.36	83.69	83.30	85.30
Hospitalization (%)					
Yes	9.06	11.29	11.66	13.07	9.79
No	90.94	88.71	88.34	86.93	90.21
Density of hospital beds in urban areas (per 1000 population) (mean)	72.13	80.77	86.07	91.24	94.40
Density of hospital beds in rural areas (per 1000 population) (mean)	31.74	35.31	39.12	45.19	48.71
Density of medical professionals in urban areas (per 1000 population) (mean)	85.17	96.94	103.84	109.60	118.10
Density of medical professionals in rural areas (per 1000 population) (mean)	36.48	39.26	42.55	47.42	52.52

*RMB: 10 000 Chinese Renminbi, about US$ 1500 and minimum value excluding zero

**Fig. 4:**
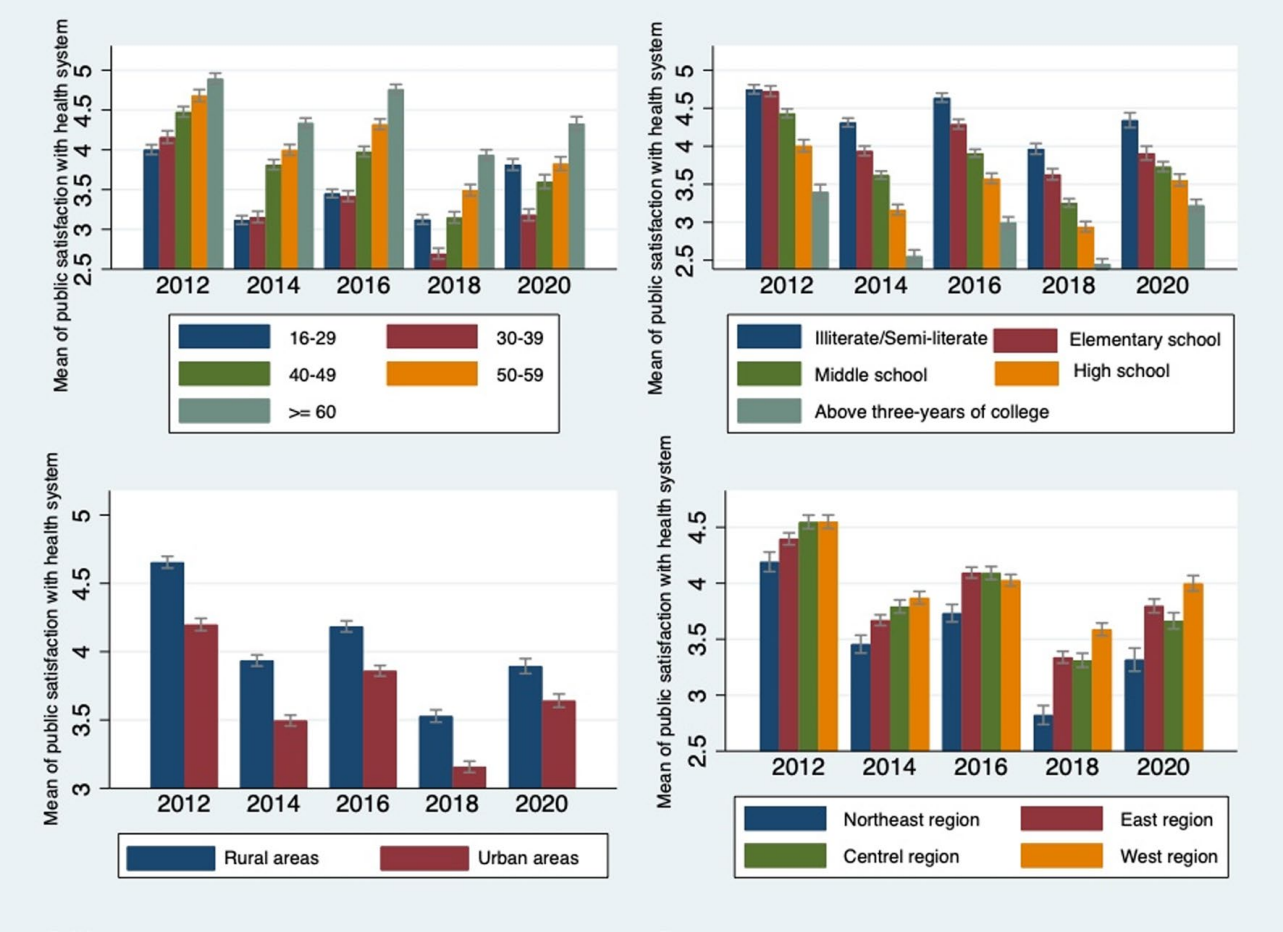
Mean public satisfaction score by socio-demographic characteristics

**Fig. 5:**
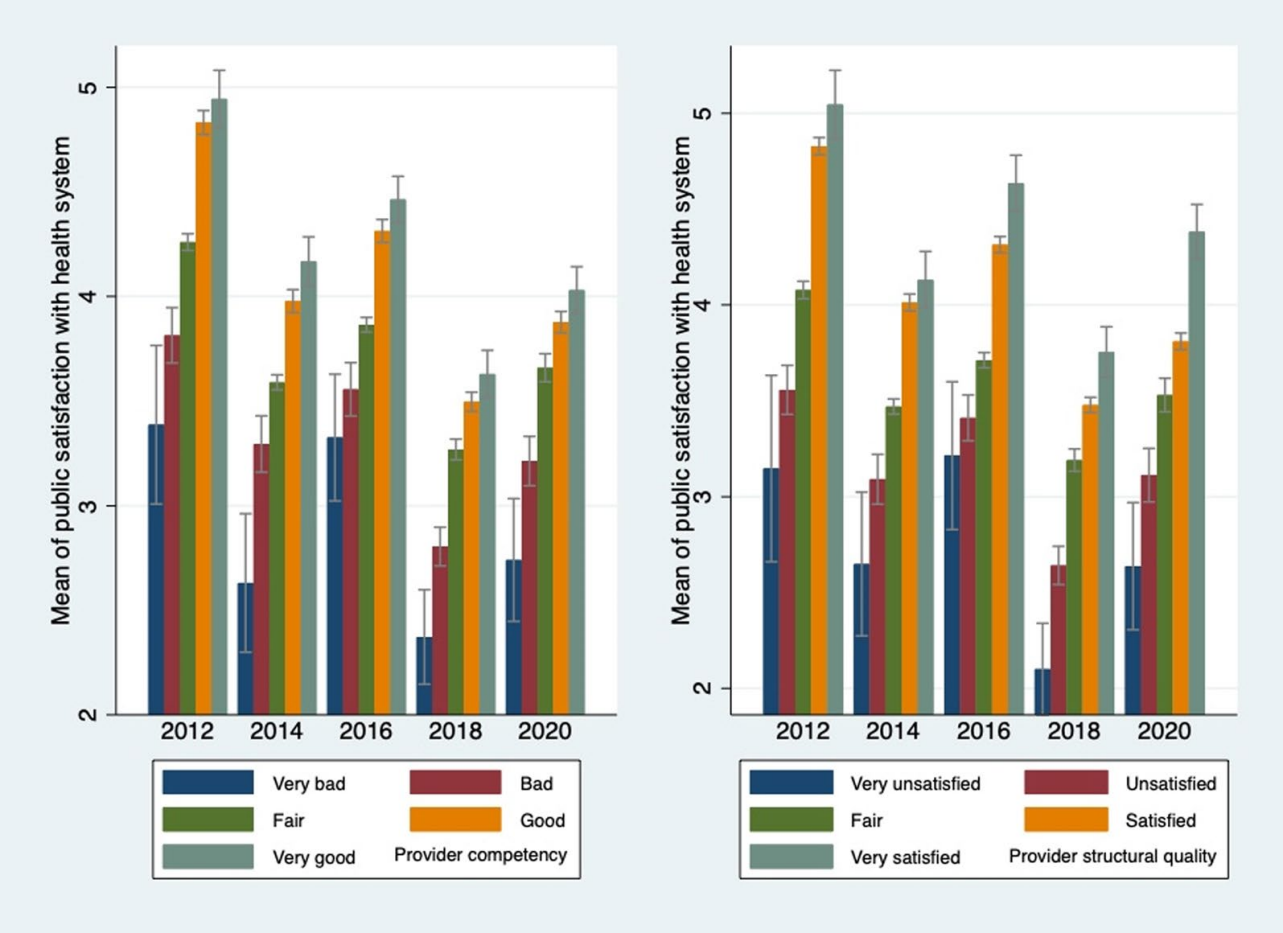
Mean public satisfaction score by perceived quality of care

Figure 4 presents the mean public satisfaction score by socio-demographic characteristics. Young people, people with higher level of educational attainment, urban respondents, and people who live in the northeast region reported relatively lower level of public satisfaction with the health system. One-way analysis of variance (ANOVA) showed significant differences in public satisfaction across age groups, educational attainment, place of residence and geographical location of respondents (data not shown). Figure 5 shows the mean public satisfaction score by perceived quality of care. Individuals who evaluated the provider competence and provider structural quality as high had a high level of public satisfaction with the health system. One-way ANOVA showed significant differences in public satisfaction across provider competence and provider structural quality (data not shown).

The results of the two-way fixed-effects ordered logistic regression analysis are reported in Table 2. The results revealed that rural respondents aged 30–39 years and 50–59 years were less likely to be satisfied with the health system compared with rural respondents in the 16–29 years age group (OR 0.90, 95% CI 0.80–1.00; OR 0.82, 95% CI 0.68–1.00). Compared with illiterate or semi-literate people, people with more than 3 years of college education were less likely to be satisfied with the health system in rural areas (OR 0.69, 95% CI 0.49–0.95). Rural respondents with high incomes had higher probability of reporting being satisfied, although the odds ratio is close to 1.00 (OR 1.01, 95% CI 1.00–1.03). The density of medical professionals (per 1000 population) had a positive association with public satisfaction in rural areas, with the odds ratio again being close to 1.00 (OR 1.01, 95% CI 1.00–1.02).

**Table 2:** Two-way fixed-effects ordered logistic regression analysis on public satisfaction with the health system

	Urban	Rural
	Odd ratios (95% CI)	Odd ratios (95% CI)
Provider competence		
Very bad (ref.)	1.00	1.00
Bad	1.03 (0.83–1.27)	1.16 (0.98–1.38)
Fair	1.17 (0.95–1.44)	**1.26 (1.07–1.48)**
Good	**1.27 (1.03–1.56)**	**1.37 (1.16–1.61)**
Very good	**1.36 (1.09–1.69)**	**1.30 (1.09–1.55)**
Provider structural quality		
Very unsatisfied (ref.)	1.00	1.00
Unsatisfied	1.23 (0.98–1.54)	1.15 (0.93–1.41)
Fair	**1.44 (1.15–1.79)**	**1.26 (1.03–1.54)**
Satisfied	**1.63 (1.31–2.03)**	**1.47 (1.20–1.80)**
Very satisfied	**1.87 (1.48–2.36)**	**1.50 (1.21–1.86)**
Age group		
16–29 (ref.)	1.00	1.00
30–39	1.03 (0.93–1.15)	**0.90 (0.80–1.00)**
40–49	0.91 (0.77–1.07)	0.86 (0.74–1.01)
50–59	0.83 (0.68–1.01)	**0.82 (0.68–1.00)**
≥ 60	0.90 (0.71–1.14)	0.84 (0.67–1.05)
Sex		
Female (ref.)	1.00	1.00
Male	1.52 (0.84–2.74)	0.65 (0.36–1.16)
Educational attainment		
Illiterate/semi-literate (ref.)	1.00	1.00
Elementary school	1.05 (0.74–1.50)	0.92 (0.74–1.15)
Middle school	0.91 (0.63–1.33)	0.84 (0.65–1.10)
High school	0.87 (0.59–1.29)	0.76 (0.57–1.01)
More than 3 years of college	0.95 (0.63–1.44)	**0.69 (0.49–0.95)**
Marital status		
Other (ref.)	1.00	1.00
Married	**0.90 (0.81–1.00)**	1.01 (0.91–1.12)
Personal income (10 000 yuan)	0.99 (0.99–1.00)	**1.01 (1.00–1.03)**
Employment status		
Other (ref.)	1.00	1.00
Employed	0.96 (0.91–1.02)	0.99 (0.95–1.04)
Medical insurance coverage		
No (ref.)	1.00	1.00
Yes	**1.09 (1.02–1.17)**	1.07 (1.00–1.15)
Locations of respondents		
Northeast region (ref.)	1.00	1.00
East region	0.69 (0.43–1.08)	0.53 (0.24–1.21)
Central region	1.07 (0.66–1.74)	0.78 (0.33–1.83)
West region	**0.55 (0.33–0.91)**	0.76 (0.33–1.72)
Self-rated health status		
Poor (ref.)	1.00	1.00
Fair	**1.10 (1.01–1.19)**	1.03 (0.96–1.10)
Good	**1.11 (1.03–1.20)**	0.98 (0.92–1.04)
Chronic conditions		
No (ref.)	1.00	1.00
Yes	0.95 (0.89–1.00)	1.00 (0.95–1.06)
Hospitalization history		
No (ref.)	1.00	1.00
Yes	**1.13 (1.06–1.20)**	**1.09 (1.03–1.15)**
Density of hospital beds (per 1000 population)^*^	**1.00 (0.99–1.00)**	1.00 (0.99–1.01)
Density of medical professionals (per 1000 population)^**^	1.00 (1.00–1.01)	**1.01 (1.00–1.02)**
Year		
2012 (ref.)	1.00	1.00
2014	**0.58 (0.55–0.61)**	**0.60 (0.57–0.63)**
2016	**0.80 (0.74–0.86)**	**0.72 (0.68–0.78)**
2018	**0.43 (0.40–0.47)**	**0.42 (0.38–0.47)**
2020	**0.68 (0.61–0.75)**	**0.57 (0.50–0.65)**
Observations	59 785	64 931

Bold indicates statistical significance, *p* < 0.05. *The density of hospital beds (per 1000 population) in urban and rural areas were used in urban and rural population. **The density of medical professionals (per 1000 population) in urban and rural areas were used in urban and rural population

In urban areas, married people were less likely to be satisfied with the health system compared with those who were never married, divorced, widowed or separated (OR 0.90, 95% CI 0.81–1.00). Compared with uninsured people, urban respondents enrolled in medical insurance had higher likelihood of reporting as being satisfied with the health system (OR 1.09, 95% CI 1.02–1.17). Individuals who lived in the urban-west region show decreased odds of reporting as being satisfied with the health system compared with those who lived in the urban-northeast region (OR 0.55, 95% CI 0.33–0.91). Urban respondents with fair or good health were more likely to be satisfied with the health system than those who reported their health being poor (OR 1.10, 95% CI 1.01–1.19; OR 1.11, 95% CI 1.03–1.20).

Individuals who experienced hospitalization in the last 12 months show a higher probability of reporting as being satisfied with the health system in urban and rural areas (OR 1.13, 95% CI 1.06–1.20; OR 1.09, 95% CI 1.03–1.15). In the years 2014, 2016, 2018 and 2020, individuals were less likely to be satisfied with the health system than in 2012 in both urban and rural areas.

Regardless of urban–rural residence, perceived quality of care was positively associated with reporting of satisfaction with the health system. Individuals who evaluated the provider competence of healthcare providers as good or very good were more likely to be satisfied with the health system. Moreover, persons who reported being satisfied with the provider structural quality were more likely to be satisfied with the health system. The odds ratios for variable ‘provider structural quality’ were found to be higher than the odds ratios for the variable ‘provider competence’.

## Discussion

China saw a decline in average public satisfaction scores from 4.45 in 2012 to 3.76 in 2020. It is interesting that several countries, such as Spain [47, 48], Greece [48] and the United Kingdom [49], also reported decline in public satisfaction with their health systems. This probably implies increasing gaps between people’s expectations and the readiness of the system to meet continuously evolving needs. In China, considerable investments in healthcare through the new health reform initiatives failed to close the gap between what the population expects from the health system and what the system is able to deliver [50]. Since individuals who are satisfied with their health system are more likely to be positive about their health situation and more likely to be engaged in their treatment, addressing public satisfaction issue is not simply related to service quality or infrastructural quality of healthcare facilities. Therefore, identifying the factors associated with lower public satisfaction will be of interest to policymakers.

Regardless of rural–urban residence, perceived quality of care was positively associated with public satisfaction. Due to a lack of formal referral structure, two in five older patients in China bypassed primary care facilities to obtain care from upper-level healthcare providers. Urban patients were nearly twice as likely as rural patients to bypass primary care centres [51]. The phenomenon of bypassing probably reflects patients’ pursuit for better quality of care. Perceived poor quality of care encourages people to look for alternative providers of healthcare, which probably increases dissatisfaction with the health system. In addition, we found that the most powerful predictor for public satisfaction with the health system was provider structural quality. This aspect was much more important than provider competence. The Chinese government has invested substantially in physical and information technology infrastructure during the new health reforms [50]. However, infrastructure construction alone is not enough to improve perceived structural quality of healthcare providers. A sustained shortage of qualified health workers at primary care facilities may be viewed as a structural problem, as physician shortage is a significant bottleneck for strengthening the healthcare delivery system [52].

This study found that individuals who experienced hospitalization were more likely to be satisfied with the health system in both urban and rural areas. Recent use of inpatient care can be considered a proxy for patients’ experiences with specialist care, and therefore, low public satisfaction is more likely to be associated with experiences with primary care providers rather than specialists. A study shows that 95% of Chinese patients viewed their inpatient care experiences as positive [53]. A better patient experience is related to higher level of public satisfaction [19].

We found that public satisfaction was higher among rural respondents, possibly because of their lower expectations from the health system [26]. Additionally, the health system reform since 2009 affirmed the government’s role in financing healthcare and redistributing finance and human health resources towards the rural areas [1]. Rural residents reported higher level of satisfaction compared with their urban counterparts after the implementation of health system reform that increased public funding for the rural health system [54]. In rural areas, individuals report lower likelihood of being satisfied with the health system with increasing age of the individual. Older adults are more likely to have multiple morbidities and these specialized services may not be widely available in rural areas [55]. Unmet health needs negatively affect public satisfaction [56].

We found that individuals with more than 3 years of college education showed decreased odds of reporting public satisfaction with the health system in rural areas. Again, this relationship may be explained by the changing expectations from the system with increasing educational attainment. Rural respondents with a higher level of education had a higher level of expectation [11, 19]. Higher expectations lead to lower public satisfaction with the health system. Moreover, rural residents with higher education have lower loyalty towards primary care facilities in rural areas, meaning that they tend to bypass primary care facilities to seek healthcare in urban hospitals [57]. The barriers rural residents face to seek care from upper-level facilities, such as travel cost and time, cost of hospital registration and accessing inpatient care, may have affected public satisfaction with the health system adversely. After controlling for educational attainment, higher personal income showed a higher probability of reporting public satisfaction in rural areas. Income is a core socio-economic status indicator, and higher income may improve access to healthcare services. Individuals with lower incomes in rural areas often have to depend on the healthcare services available in their area. In rural areas, health-related resources are in short supply, which may cause a low level of public satisfaction [27]. One measure of healthcare personnel availability is the density of medical professionals, and in rural areas it shows a positive association with public satisfaction. Patient experience is significantly associated with public satisfaction [19]. With increasing density of medical professionals in rural areas, the healthcare provider may be able to spend more time with patients improving patient experience as well as public satisfaction [58].

People enrolled in medical insurance schemes were more likely to be satisfied with the health system compared with the uninsured people in urban areas. Medical insurance schemes reduce out-of-pocket medical expenses and lower out-of-pocket costs reduce financial barriers to access. Previous studies show that financial barriers are associated with reduced public satisfaction [13, 58]. Moreover, urban respondents who self-reported their health as fair or good reported a higher probability of satisfaction. This is not unexpected, as healthy individuals use fewer health services and are less likely to use specialized services, which probably helps to improve the degree of satisfaction with the system [10].

We found that married people were less likely to be satisfied with the health system in urban areas. However, a previous study in South Korea reported that people who are married or single were more likely to be satisfied with the health system [59]. In China, it is possible that affordability of healthcare is a significant concern for married individuals compared with others [60, 61]. Individuals who lived in the urban-west region were less likely to be satisfied with the health system than those who lived in the urban-northeast region. The regional differences in public satisfaction are probably because of differences in healthcare resources availability across the regions. The urban-west region (China’s underdeveloped areas) is less attractive to qualified hospital medical staff, and thus the health system lacks the capacity to respond to local needs [62]. Additionally, poor regional economic performance may directly affect the citizens’ perception and lead to a poor perception of public policies, including health-related policies, which negatively affect public satisfaction [11].

### Limitations

Several limitations of the study should be noted. First, this study assessed public satisfaction using a self-reported Likert scale rating. The answers depend particularly on factors not fully understood, for example, recent media coverage of scandals about healthcare [12]. Second, accounting for time-varying unobserved heterogeneity and simultaneity bias in this study, we cannot draw conclusions about causal inferences. Last, the data were obtained via face-to-face or telephone interviews, and thus, the limitations of all self-reported data exist, such as recall bias and the unreliability of responses when respondents are under pressure.

## Conclusions

Our analysis found that average public satisfaction score decreased significantly over the years 2012–2020 despite improvements in physical infrastructure and knowledge/expertise of healthcare providers. We found that perceived good quality of care and hospitalization showed a higher probability of reporting public satisfaction regardless of rural–urban residence. Older adults and individuals with more than 3 years of college education were less likely to be satisfied with the health system in rural areas. Moreover, personal income and the density of medical professionals had a positive association with public satisfaction in rural areas. Having medical insurance coverage and individuals with fair or good self-rated health had a higher probability of reporting public satisfaction in urban areas. In addition, we found that married people and individuals who lived in the urban-west region were less likely to be satisfied with the health system than others.

Improving public satisfaction with the health system is an important policy issue for China because the degree of responsiveness of the system to the needs of the population is an important measure of health system performance. This study has identified several factors affecting public satisfaction and some of the factors are amenable to policy changes. First, the health delivery system of China needs to be rebranded as the provider of high-quality healthcare services. Second, the health system may consider improving access to healthcare services for vulnerable population groups, such as the poor and the elderly in rural areas. Third, the Chinese government should continue to strengthen the rural healthcare workforce by increasing the enrolment of rural students and providing free or subsidized medical education for them. Last, since high out-of-pocket expenses adversely affect public satisfaction in health system, expanding medical insurance to achieve universal coverage with built-in protection against catastrophic health expenditures should improve public satisfaction significantly.

## Data Availability

The dataset used for drafting the paper is a publicly available dataset available in the Peking University Open Research Data Platform repository. The dataset is downloadable for research purposes through the link: https://opendata.pku.edu.cn/dataset.xhtml?persistentId=doi:10.18170/DVN/45LCSO.

## Abbreviations

CFPS: China Family Panel Studies
ORs: Odds ratios
CIs: Confidence intervals
ANOVA: Analysis of variance
